# A Bifidobacterium Strain with Antibacterial Activity, Its Antibacterial Characteristics and In Vitro Probiotics Studies

**DOI:** 10.3390/microorganisms13061190

**Published:** 2025-05-23

**Authors:** Jing Ji, Tiange Li, Baoying Ma, Runzhong Wang

**Affiliations:** 1 School of Environmental Science and Engineering, Tianjin University, Tianjin 300072, China; 2022214062@tju.edu.cn (T.L.); skymby1999@163.com (B.M.); 2022214002@tju.edu.cn (R.W.); 2 Haihe Laboratory of Sustainable Chemical Transformations, Tianjin 300192, China; 3 Frontiers Science Center for Synthetic Biology, Tianjin University, Tianjin 300072, China

**Keywords:** probiotics, *Bifidobacterium*, antimicrobial proteins, antibacterial characteristics

## Abstract

The search for natural antimicrobials has intensified with rising food safety demands. This study evaluated 23 probiotic strains, identifying *Bifidobacterium* sp. strain TF04 as a potent inhibitor against pathogens, with inhibition zone diameters of 12.85 ± 0.12 mm (*Escherichia coli*), 14.85 ± 0.10 mm (*Staphylococcus aureus*), and 17.50 ± 0.23 mm (*Staphylococcus epidermidis*). Preliminary analysis shows that the main antibacterial compounds produced by TF04 in the process of bacterial growth inhibition are antibacterial active proteins. TF04 exhibits optimal bacteriostatic activity within the pH range of 2–4, with a notable decline in effectiveness as the pH value increases. At the same time, the bacteriostat produced by TF04 showed strong thermal stability and ultraviolet stability. TF04 demonstrated excellent probiotic potential: surviving acidic (pH 2.0, >45% viability) and bile conditions (3% bile salts, >55% survival). It showed strong auto-aggregation (40.10%) and hydrophobicity (>30%), indicating gut colonization potential, along with notable antioxidant capacity. Safety was confirmed by absent hemolytic and gelatinase activities. These properties position TF04 as a promising multifunctional candidate for food preservation, combining antimicrobial efficacy with probiotic benefits. Further studies will purify its bioactive compounds and validate applications in food systems.

## 1. Introduction

Probiotics, a category of advantageous and live microorganisms, establish residence in the human intestinal and reproductive tracts [1]. These probiotics are typically isolated from the intestinal tract or feces of healthy individuals (infants, breast milk, centenarians) and partly isolated through extraction and isolation from foods that have been safely consumed over extended durations or from fermented foods.

Probiotics are divided into traditional probiotic lactic acid bacteria, non-lactic acid bacteria probiotics, and second-generation probiotics based on their metabolic profile and source [2]. Traditional probiotic lactic acid bacteria include *Lactobacillus*, *Bifidobacterium*, *Lactococcus*, and *Enterococcus* [3]. Non-lactic acid bacteria probiotics, including *S. boulardii* and *Bacillus coagulans*, are known for their extensive history of safe use and established beneficial effects [4]. Second-generation probiotics, including *Akkermansia muciniphila*, *Bacteroides xylanisolvens*, and *Faecalibacterium prausnitzii*, represent a cluster of microorganisms challenging to culture due to their therapeutic potential in addressing specific diseases [5].

In a comprehensive systematic mutagenesis study, Gao [6] employed physical and chemical methods to mutagenize and screen *Lactobacillus acidophilus* NX2-6. Through exposure to 60Co gamma rays, performing DES mutagenesis, and carrying out protoplast UV mutagenesis, three high-yield bacteriocin strains, namely γ56, D47, and UVl56, were successfully isolated. The bacteriocin produced by *Lactobacillus bulgaricus* C-M2 demonstrated remarkable heat and pH stability. Even after being sterilized at 121 °C for 15 min, it still retained 82.1% of the antibacterial activity and maintained 85.6% of the antibacterial activity at pH 6. Upon treatment with a variety of proteases, the antibacterial activity was completely lost; however, the bacteriocin exhibited high resistance to lipase and amylase [7]. Another study [8] has shown that when lactic acid bacteria enter the host intestinal tract, lactic acid bacteria engage in competitive interactions with pathogenic bacteria, establishing colonization with mucosal epithelial cells. This process leads to a decrease in the population of harmful microorganisms, thereby lowering the risk of disease and promoting overall health. In a separate study, Bhat et al. [9] discovered that *Lactobacillus fermentum* MTCC-5898 has the ability to safeguard the barrier function of human intestinal Caco-2 cells by modulating the distribution of ZO-1 and Claudin-1 proteins. Merghni et al. [10] reported that *Lactobacillus casei* is capable of producing extracellular polysaccharides, which inhibit the adhesion of *Staphylococcus aureus*, thereby exerting an antibacterial effect. In a study by Huang et al. [11], *Bifidobacterium lactis* HN019 and *Lactobacillus acidophilus* NCFM were singled out among four probiotic strains, and their antibacterial activity against halitosis-related pathogens was explored. The study validated the application potential of *Bifidobacterium lactis* HN019 and *Lactobacillus acidophilus* NCFM in halitosis management. Cizeikiene [12] conducted an assessment of the antibacterial activity and probiotic attributes of lactic acid bacteria and bifidobacteria, with a focus on their potential applications in functional food/feed products. The antimicrobial activity of 12 strains of pathogenic bacteria such as *Staphylococcus aureus*, *Escherichia coli*, *Staphylococcus chromophilus*, and *Staphylococcus hyicus* was evaluated by agar dispersion method and broth inhibition method. The metabolites produced by *Lactobacillus paracasei* DSM 4905 and *Lactobacillus garvieae* DSM 20077 inhibited the growth of all tested pathogens. Moreover, the probiotic strains exhibited in vitro characteristics such as resistance to low pH and bile salts. Additionally, *Lactobacillus rhamnosus* YT showed a remarkable antibacterial effect. The antibacterial activity of *Lactobacillus rhamnosus* YT increased as the cells proliferated, reaching its peak during the stationary growth phase. When co-cultured with *Lactobacillus rhamnosus* YT, the biofilm formation by *Bacillus subtilis* and *Streptococcus coli* decreased. The antibacterial activity of *Lactobacillus rhamnosus* YT differed according to various culture conditions (including carbon source, nitrogen source, medium pH, and culture temperature) and antibacterial temperature [13].

Fruits and vegetables play a vital role in human nutrition and health. Nevertheless, their vulnerability to microbial spoilage renders them highly perishable. Foodborne pathogens present a significant challenge for consumers, food companies, and food safety. The utilization of synthetic preservatives in current practices carries potential health risks for consumers. Consequently, there is a growing interest in probiotic bacteriostats as food additives for food preservation applications. Probiotic inhibitors effectively manage and regulate microbial proliferation, thereby mitigating biological and chemical spoilage processes that contribute to the deterioration of color, flavor, lipids, and vitamins through natural oxidation. Bacteriocins, which are peptides or ribosomal proteins produced by bacteria, inhibit or kill other bacteria [14]. The use of bacteriocins in foods not only enhances flavor but also helps in maintaining freshness [15]. Several bacteriocins, such as Nisin, Enterocin AS-48, Oxytetracycline HC5, Peppermint, Enterocin 416K1, and Bacteriocin Bificin C6165, have been applied for food preservation [16]. Lactic acid bacteria have been designated as generally recognized as safe (GRAS) by the U.S. Food and Drug Administration (FDA), the Qualified Presumption of Safety (QPS), and the European Food Safety Authority (EFSA). Consequently, the bacteriostatic agents generated by probiotics are gaining increasing recognition as natural food preservatives.

Probiotic bacteriostatic agents find applications in various food categories, such as fruits and vegetables, chilled meat, aquatic products, and dairy products. In addition, probiotics are used in medical and feed applications. Lactic acid bacteria can inhibit intestinal pathogens and reduce the risk of related diseases. Concurrently, they can enhance gastrointestinal motility, promote food digestion, and enhance immune responses [17,18]. With consumers placing growing emphasis on food safety and developing a more negative perception of antibiotic resistance, there is an escalating interest in the development of safe and non-toxic biological preservatives. Therefore, it is crucial to find new natural bacteriostatic agents with broad-spectrum antibacterial activity and high stability for food preservation and other fields. Previous research experiments have established the safety and harmlessness of probiotics, highlighting their potential to evolve into natural bacteriostatic agents.

This study focuses on the systematic screening of 23 probiotic strains preserved by Tianda Tianfu Biotechnology Co., Ltd., (Hong Kong) to identify highly effective antibacterial strains. Further investigations delved into the bacteriostatic attributes of the generated bacteriostatic agents, coupled with an analysis of the potential antibacterial mechanisms. These findings aim to furnish a theoretical foundation for the isolation, purification, and utilization of natural antibacterial agents derived from probiotics.

## 2. Materials and Methods

### 2.1. Screening of Probiotics with Bacteriostatic Activity

The activated probiotics were inoculated into MRS broth at a 5% (*v*/*v*) inoculation amount, cultured at 37 °C for 24 h, and centrifuged at 8000 r/min for 15 min to obtain supernatant and bacterial sludge. The supernatant was filtered by 0.45 μm microporous membrane, while the bacterial sludge was added with normal saline to make a suspension. The antibacterial properties were determined with *Escherichia coli* ATCC 25922, *Staphylococcus aureus* ATCC 12600, and *Staphylococcus epidermidis* ATCC 8032 as indicator strains.

The viable counts (CFU/mL) of probiotic strains were determined by serial dilution in sterile PBS, followed by plating on MRS agar and incubation under appropriate conditions. The concentration of the indicator strain was set at 10^7^ CFU/mL. A volume of 200 μL of pathogenic bacterial liquid was evenly spread on an agar plate using a spreading rod. Subsequently, holes were created on the agar surface using an Oxford cup (Shanghai Precision Instrument Co., Ltd., Shanghai, China). To these holes, 100 μL MRS liquid medium (blank control group), 100 μL of bacterial sludge suspension and 100 μL of bacterial liquid were added. After allowing the agar medium to absorb the liquids, the plates were anaerobically incubated at 37 °C for 48 h. Following incubation, the diameter of the inhibition zone was measured using a vernier caliper (Huayang Bio-Technology Co., Ltd., Huayang, China) and recorded.

### 2.2. Strain Identification

DNA extraction was performed according to the instruction program of Bacterial Fungal Genome Extraction Kit (Beijing Liuhe BGI Co., Ltd., Beijing, China). Subsequently, amplification was performed using primers 27F(AGAGTTTGATCCTGGCTCAG) and 1492R(TACGGCTACCTTGTTACGACTT). PCR was performed in a 25 µL reaction system consisting of 21 µL of BGI 2× Super PCR Mix (Beijing Liuhe BGI Co., Ltd., Beijing, China), 1 µL Primer F (5p), 1 µL Primer R (5p), and 2 µL of template. PCR amplification protocol consisted of an initial denaturation step at 96 °C for 35 min, followed by 35 cycles of denaturation at 96 °C for 30 s, annealing at 56 °C for 30 s, extension at 72 °C for 1 min, and a final extension at 72 °C for 5 min. A volume of 3 µL of the PCR product was subjected to analysis on a 1.0% agarose gel for band visualization. Subsequently, the PCR product was purified using a standard magnetic bead purification procedure. This method exploits the capability of magnetic beads to adsorb or release charged materials, thereby enabling the adsorption of DNA in a high-salt, low pH solution and the subsequent release of DNA in a low-salt, high-phosphate solution, facilitating the isolation and purification of the DNA product. The purified PCR products were sequenced by an online assay, and the sequences of the isolated yeasts were identified at the species level by NCBI BLAST web suite (https://www.ncbi.nlm.nih.gov/). The identified strains were subjected to molecular evolutionary analysis by constructing evolutionary trees using the neighbor-joining method in MEGA-X software.

### 2.3. Determination of Strain Growth Curves

The activated probiotics were inoculated in MRS broth and cultured at 37 °C. The optical density (OD600) was measured at 4 h intervals over a 48 h period (i.e., 0, 4, 8, 12, 16, 20, 24, 28, 32, 36, 40, 44, and 48 h). A growth curve of the strain was constructed with the OD600 values on the y-axis and the culture time on the x-axis.

### 2.4. Experiment on the Exclusion of Organic Acids

The antibacterial activity attributed to organic acids generated during probiotic fermentation was investigated. To determine whether the antibacterial substance is an organic acid, the pH of distilled water was adjusted to the same as the bacteriostatic agent by using lactic acid and acetic acid with concentrations of 15% and 20%, respectively. The antibacterial activity was determined by punching method with *Escherichia coli*, *Staphylococcus aureus*, and *Staphylococcus epidermidis* (10^7^ CFU/mL) as indicator bacteria.

### 2.5. Experiment on the Exclusion of Hydrogen Peroxide

The antibacterial activity associated with hydrogen peroxide produced during probiotic fermentation was investigated. To determine whether the bacteriostatic agent contains hydrogen peroxide, 1 mL of 10 mg/mL peroxidase was mixed with 1 mL of bacteriostatic agent, while the untreated bacteriostatic agent was used as the control. The antibacterial agent was treated in a water bath pot at 37 °C for 24 h. The antibacterial activity was determined by punching method with *Escherichia coli*, *Staphylococcus aureus*, and *Staphylococcus epidermidis* (10^7^ CFU/mL) as indicator bacteria.

### 2.6. Determination of Protease Sensitivity

Papain, Alkaline protease, Protease K, Pepsin, and Trypsin with a final concentration of 1.0 mg/mL were added to the bacteriostatic agent, respectively. After 2 h of constant temperature water bath at 37 °C, the samples were subsequently subjected to a 10 min incubation at 80 °C to halt enzyme activity. The supernatant culture medium without protease treatment served as a control. The antibacterial activity was determined by punching method with *Escherichia coli*, *Staphylococcus aureus*, and *Staphylococcus epidermidis* (10^7^ CFU/mL) as indicator bacteria.

### 2.7. Stability Test of Bacteriostatic Agent

#### 2.7.1. Determination of Acid-Base Stability

The bacteriostatic agent’s pH was adjusted to values of 2, 4, 6, 7, 8, 9, and 10 by adding appropriate amounts of HCl and NaOH. Subsequently, the antibacterial activity at different pH was determined by punching method with *Escherichia coli*, *Staphylococcus aureus*, and *Staphylococcus epidermidis* (10^7^ CFU/mL) as indicator bacteria.

#### 2.7.2. Determination of Thermal Stability

The antibacterial agent was heated at 50 °C, 60 °C, 70 °C, 80 °C, 90 °C, and 100 °C water bath for 30 min. Antibacterial activity at different temperatures was determined by punching method with *Escherichia coli*, *Staphylococcus aureus*, and *Staphylococcus epidermidis* (10^7^ CFU/mL) as indicator bacteria.

#### 2.7.3. Determination of Ultraviolet Stability

The antibacterial agent was placed in the ultraviolet irradiation box for 2 h, 4 h, 6 h, 8 h, and 10 h, respectively. Subsequently, the antibacterial activity at different UV irradiation was determined by punching method with *Escherichia coli*, *Staphylococcus aureus*, and *Staphylococcus epidermidis* (10^7^ CFU/mL) as indicator bacteria.

### 2.8. Probiotic Analysis

#### 2.8.1. Resistance to Low pH

The pH of MRS medium was adjusted to 1.0, 2.0, 3.0, 4.0, and 5.0. Then, the activated strains were inoculated into the MRS medium with different pH at 3% (*v*/*v*) and cultured at 37 °C for 5 h. After treatment, bacterial survival rates were quantified via serial dilution and plate counting. Briefly, cultures were diluted 10-fold serially in sterile PBS, plated on MRS agar, and incubated at 37 °C for 24 h. The untreated bacterial strain was used as the control, and colonies numbering between 30 and 300 were selected for valid counting.

#### 2.8.2. Resistance to Bile Salts

MRS liquid medium with different concentrations of bile salts (0%, 1.0%, 2.0% and 3.0%) were prepared. Subsequently, the activated strains were inoculated into the MRS medium with different concentrations of bile salts at 3% (*v*/*v*) and cultured at 37 °C for 4 h. Then, cultures were diluted 10-fold serially in sterile PBS, plated on MRS agar, and incubated at 37 °C for 24 h. The untreated strain was used as a control. The colony counts were within the range of 30–300 colonies for counting.

Calculate the survival rate with the following formula:

$$\mathrm{Survival}\;\mathrm{rate}=V_{1}/V_{0}\times 100\%$$

*V*_1_ represents the number of viable bacteria in the tolerant medium and *V*_0_ denotes the number of viable bacteria in the MRS medium without any additional components.

#### 2.8.3. Auto-Aggregation Ability and Hydrophobicity

The strains were inoculated in MRS liquid medium and cultured at 37 °C for 24 h, then centrifuged at 6000 rpm for 5 min in a high-speed centrifuge to collect the cells. The cells were washed three times with PBS and resuspended to OD600 = 0.6. The initial absorbance value (*Ab*_0_) was recorded. The cells were placed at 37 °C and the OD600 (*Ab_t_*) of the supernatant was measured every 2 h.

The Auto-aggregation ability was calculated with the following formula:

$$\mathrm{Auto}-\mathrm{aggregation}\;\mathrm{Ability}=\left(Ab_{0}-Ab_{t}\right)/Ab_{t}\times 100\%$$

The strains were inoculated in MRS liquid medium and cultured at 37 °C for 24 h. Following incubation, the culture was centrifuged at 6000 rpm for 5 min using a high-speed centrifuge to harvest the cells. The cells were washed three times with PBS and then resuspended to an OD600 of 0.7. The initial absorbance (*Ab_i_*) was recorded. The bacterial suspension was mixed with toluene or xylene in a ratio of 3:1. The aqueous phase was separated by incubation for 70 min at 37 °C in a constant temperature incubator, and its absorbance was detected at 600 nm (*Ab_f_*).

The hydrophobicity was calculated with the following formula:

$$\mathrm{Hydrophobicity}=\left(Ab_{i}-Ab_{f}\right)/Ab_{i}\times 100\%$$

#### 2.8.4. Hemolytic Activity

The strains were inoculated on the Colombian blood agar plate and cultured at 37 °C for 24 h. The presence of a clear transparent zone around the colony was observed. A grass green ring indicated α hemolysis, while a completely transparent hemolysis ring surrounding the colony signified β hemolysis. The absence of any change in the culture medium around the colony indicated γ hemolysis, representing no hemolysis. *Staphylococcus aureus* was used as a positive control.

#### 2.8.5. Gelatinase Activity

The strains were inoculated in gelatin basal medium at 37 °C for 24 h. *Staphylococcus aureus* was used as a positive control. After incubation, all groups were placed in a refrigerator for freezing. Subsequently, the groups were observed after a period of time to determine if liquefaction of the inoculated strains occurred. A positive result was indicated by liquefaction in the test tube inoculated with the strain, while coagulation denoted a negative result.

#### 2.8.6. DPPH Reduction Activity

The DPPH clear ability of fermentation supernatant and bacterial suspension was determined. Sample group ($A_{s}$) consisted of 2 mL fermentation supernatant/bacterial suspension mixed with 2 mL of 0.0004 mol/L DPPH solution. Blank group ($A_{b}$) involved replacing the DPPH solution in the $A_{s}$ group with anhydrous ethanol. Control group ($A_{p}$) entailed substituting the fermentation supernatant or bacterial suspension in the $A_{s}$ group with MRS liquid medium. All groups were incubated in the dark at 37 °C for 30 min. The absorbance value A at 517 nm was measured.

$$\mathrm{DPPH}\;\mathrm{reduction}\;\mathrm{activity}=\left(1-\frac{A_{s}-A_{b}}{A_{p}}\right)\times 100\%$$

#### 2.8.7. Hydroxyl Radical Reduction Activity

The scavenging ability of bacterial solution to hydroxyl radicals was determined by Fenton reaction system. Control group ($A_{p}$) comprised 1 mL brilliant green (0.435 mmol/L), 1 mL FeSO_4_ (2.5 mmol/L), 1 mL H_2_O_2_ (3.0%), 1 mL ddH_2_O, and 1 mL PBS. Blank group ($A_{b}$) involved replacing H_2_O_2_ in $A_{p}$ group with ddH_2_O_2_. Sample group ($A_{s}$) entailed substituting ddH_2_O in $A_{p}$ group with fermentation supernatant or bacterial suspension. All groups were incubated in the dark at 37 °C for 30 min. The absorbance value A at 525 nm was measured.

$$\mathrm{Hydroxyl}\;\mathrm{radical}\;\mathrm{reduction}\;\mathrm{activity}=\frac{A_{s}-A_{p}}{A_{b}-A_{p}}\times 100\%$$

#### 2.8.8. Statistical Analysis

All experiments were performed in triplicate, and the results were expressed as mean ± standard deviation (SD). Data analysis involved one-way analysis of variance (ANOVA), followed by the Tukey test to indicate statistically significant differences (*p* < 0.05).

## 3. Results

### 3.1. Screening of Probiotics with Bacteriostatic Activity

**Figure 1:**
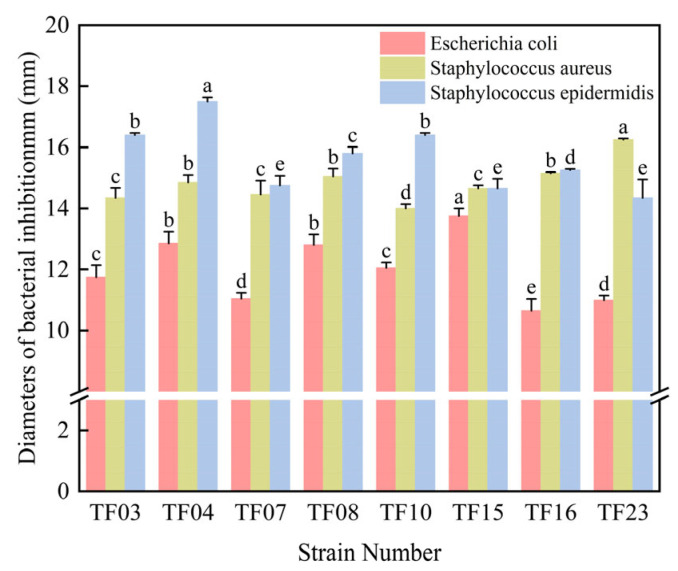
Screening of probiotics with bacteriostatic activity. The superscript letters (a, b, c, d, e) indicate statistically significant differences among treatments (*p* < 0.05, as determined by one-way ANOVA followed by Tukey’s post hoc test).

The pre-activated probiotic strains (23 strains) were inoculated into MRS liquid medium at 5% (*v*/*v*) inoculation volume and cultured at 37 ± 0.5 °C for 24 h. Following incubation, the cultures were centrifuged at 8000 rpm for 15 min. The obtained cell-free supernatant (CFS) was filtered through 0.45 μm sterile microporous membranes, whereas the bacterial pellet was resuspended to prepare a bacterial suspension. The antimicrobial activity against *Escherichia coli*, *Staphylococcus aureus*, and *Staphylococcus epidermidis* was evaluated using the agar well diffusion assay. The experimental results showed that the pH of the fermentation supernatant of 23 strains of probiotics preserved in the laboratory was about three. Among these, eight strains had strong inhibitory effects on *Escherichia coli*, *Staphylococcus aureus*, and *Staphylococcus epidermidis* (Figure 1).

The antibacterial effects of different probiotic strains varied significantly against the three indicator strains. Generally, the inhibitory activity against *Escherichia coli* was the weakest, whereas stronger effects were observed against *Staphylococcus aureus* and *Staphylococcus epidermidis*. This discrepancy may be attributed to the fact that *E. coli* is a Gram-negative bacterium, whereas *S. aureus* and *S. epidermidis* are Gram-positive.

Among the eight probiotic strains evaluated, TF15, TF04, and TF08 exhibited superior inhibitory effects against *E. coli*, with inhibition zone diameters of 13.75 ± 0.24 mm, 12.85 ± 0.21 mm, and 12.80 ± 0.17 mm, respectively. Meanwhile, TF23, TF16, TF08, and TF04 manifested robust antibacterial activity against *S. aureus*, producing inhibition zones of 16.25 ± 0.19 mm, 15.15 ± 0.23 mm, 15.05 ± 0.24 mm, and 14.85 ± 0.21 mm, respectively. Notably, TF04 displayed the highest inhibitory efficacy against *S. epidermidis*, with an inhibition zone diameter reaching 17.50 ± 0.18 mm.

Comprehensive assessment revealed that strain TF04 exhibited the most potent antimicrobial metabolite production among all tested strains. Therefore, TF04 was selected for further investigation

### 3.2. Strain Identification

**Figure 2:**
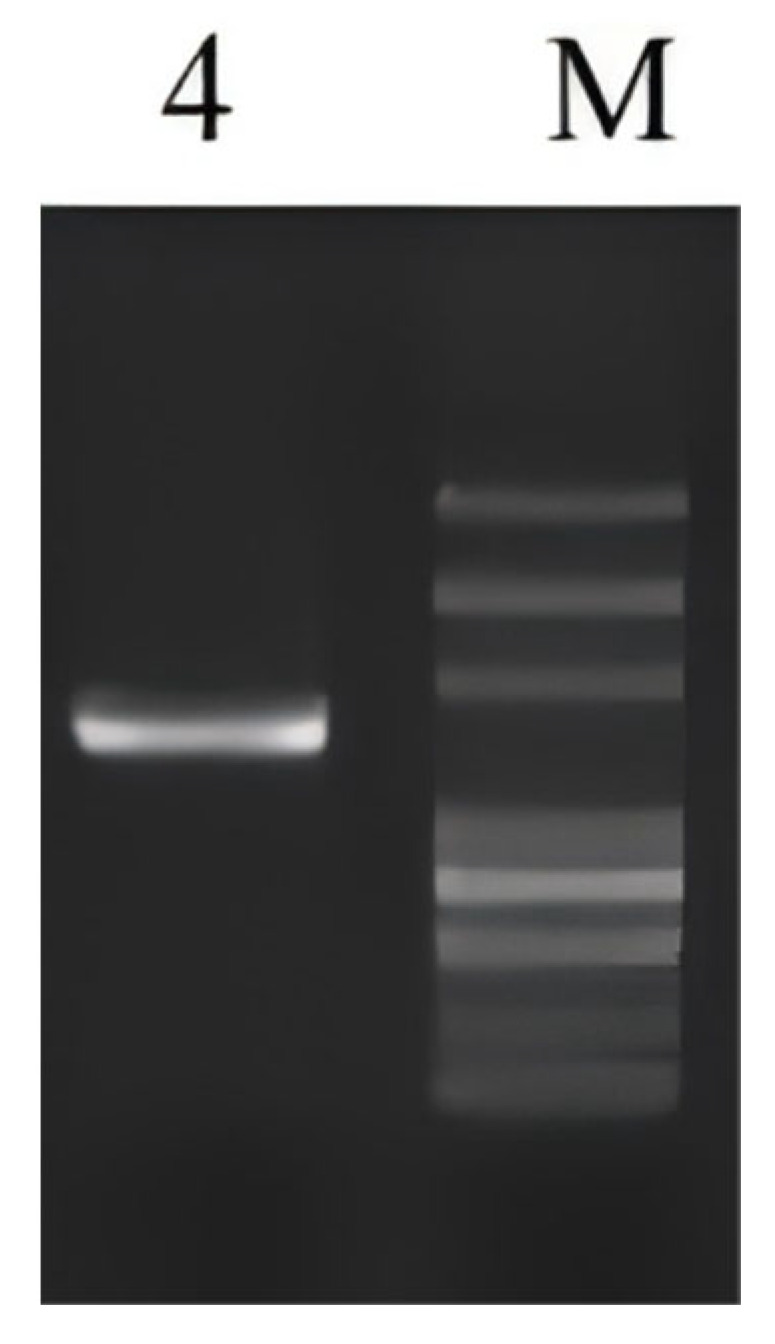
Results of agarose gel electrophoresis. Lane 4 represents probiotic strain TF04. The DNA marker (M) lanes, from top to bottom, correspond to 5000, 3000, 2000, 1000, 750, 500, 250, and 100 bp, respectively.

The 16S rDNA gene was amplified via PCR using universal primers 27F/1492R. Following amplification, the single-band PCR products were purified in accordance with magnetic bead-based DNA purification protocols. The purified amplicons were subsequently subjected to automated sequencing analysis. As demonstrated in Figure 2, agarose gel electrophoresis revealed PCR products within the expected size range of 1000–2000 base pairs.

**Figure 3:**
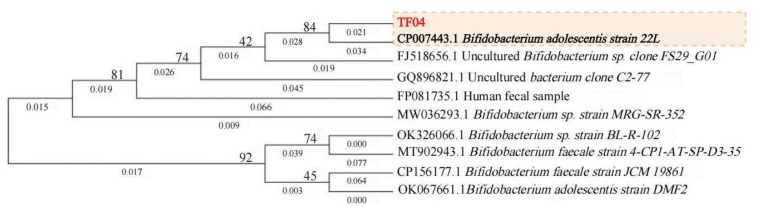
Phylogenetic tree.

The 16S rDNA gene sequence of the target strain was aligned with reference sequences in the NCBI database, revealing several strains exhibiting significant homology. Subsequent phylogenetic analysis generated an evolutionary tree (Figure 3), constructed using sequences from both the test strain and closely related reference strains obtained through comparative analysis.

Phylogenetic tree analysis demonstrated that strain TF04 showed the closest evolutionary relationship with *Bifidobacterium adolescentis* strain 22L, followed by *Bifidobacterium* sp. FS29_G01. Through 16S rRNA gene sequencing analysis, strain TF04 was identified as belonging to the genus *Bifidobacterium*. Although 16S rDNA gene analysis did not support its classification as a novel species, strain TF04 demonstrated unique antimicrobial properties. Therefore, this isolated strain has been provisionally designated as *Bifidobacterium* sp. strain TF04 (GenBank accession No. PQ637525) for subsequent functional characterization. For long-term preservation and scientific reference, this strain was designated as *Bifidobacterium jiwang* and deposited in the China General Microbiological Culture Collection Center (CGMCC) on 14 November 2024, with the accession number CGMCC No. 32629.

### 3.3. The Growth Curve of Strain TF04

**Figure 4:**
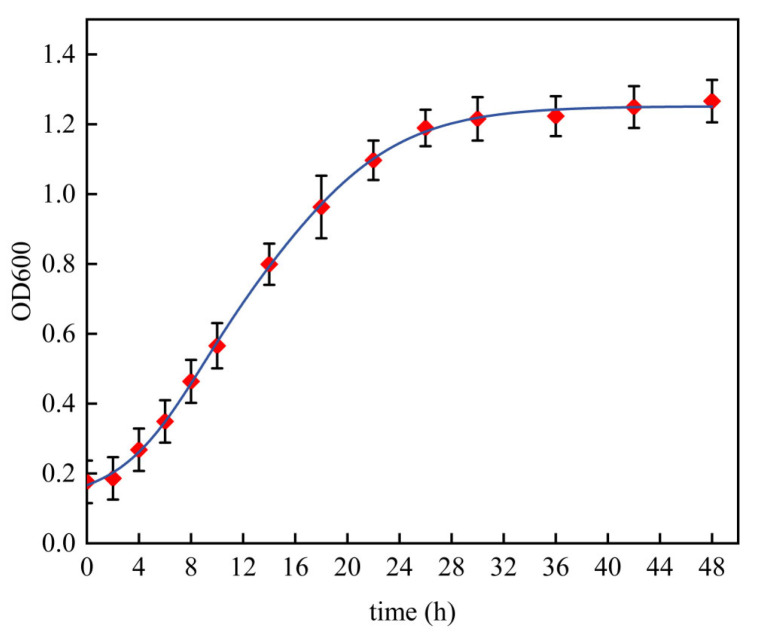
The growth curve of strain TF04.

The growth curve of TF04 is shown in Figure 4.

Following an initial 4 h incubation period, strain TF04 entered the logarithmic growth phase (OD600 = 0.268 ± 0.061), demonstrating rapid proliferation and growth. Subsequently, after 26 h of incubation, the strain transitioned into a stabilization phase (OD600 = 1.198 ± 0.052), with the final OD600 value stabilizing at around 1.220.

### 3.4. Preliminary Identification of Antimicrobial Active Substances from Strain TF04

#### 3.4.1. Exclusion of Organic Acids as Primary Antimicrobial Factors

To determine whether the observed antimicrobial activity of *Bifidobacterium* sp. strain TF04 was primarily mediated by organic acids, a systematic exclusion experiment was conducted.

During the fermentation process of probiotics, organic acids with antimicrobial properties are produced. For instance, Yang Zhan et al. [19] examined the active metabolites of *Bifidobacterium adolescentis* B9589 within its effective peak range and identified two organic acids and their derivatives. Similarly, Dou et al. [20] discovered that the fermentation products of *Bifidobacterium longum* subsp. *iuvenis* (*Bl. iuvenis*) contained glycolic acid. Based on research on the antimicrobial mechanisms of organic acids, their inhibitory efficiency has been demonstrated to be significantly associated with the acid dissociation constant (pKa). Organic acids release H+ through proton dissociation, creating a transmembrane pH gradient around bacterial cells. This results in membrane potential depolarization and inhibits ATPase activity, thereby disrupting the energy metabolism pathways of pathogenic bacteria and suppressing the growth and proliferation of acid-sensitive strains [21]. Notably, low-molecular-weight organic acids can traverse the outer membrane barrier of bacteria owing to their high lipophilicity and superior membrane permeability. Upon reaching a critical intracellular concentration, they interfere with protein biosynthesis, thereby exerting bacteriostatic effects [22]. Structural–activity relationship study indicated that the antimicrobial efficacy of organic acids is strongly correlated with their electron cloud distribution [23]. A previous research has demonstrated that acetic acid (pKa = 4.76), compared to lactic acid (pKa = 3.86) and citric acid (pKa = 3.13), exhibits more significant growth inhibition against *Listeria monocytogenes* at equimolar concentrations [22]. Building upon these findings, this study employed lactic acid (a putative probiotic metabolite) and acetic acid (a potent antimicrobial agent) to establish a dual-factor exclusion system, thereby verifying that the antibacterial activity of the strain is predominantly mediated by non-acidic metabolites.

**Figure 5:**
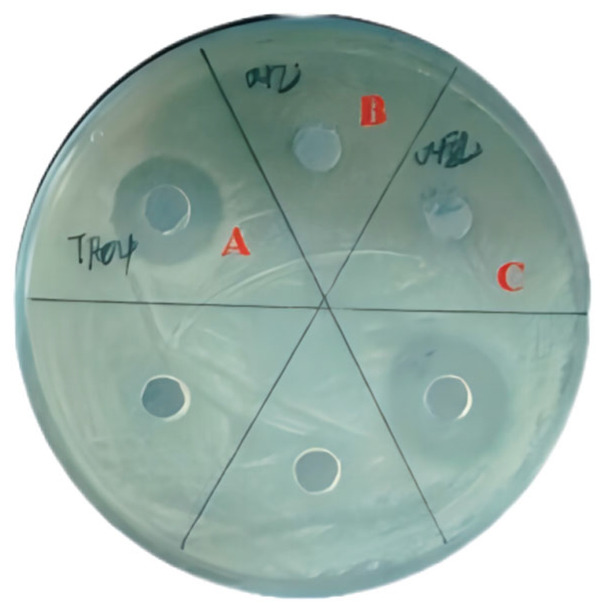
Exclusion of organic acids of inhibitory active substances of *Bifidobacterium* sp. strain TF04. Region A is the experimental results of the bacteriostatic agent produced by *Bifidobacterium* sp. strain TF04. Region B is the antibacterial properties of acetic acid. Region C is the antibacterial properties of lactic acid.

Distilled water was adjusted to pH 3.2 (matching the pH of cell-free culture supernatant of *Bifidobacterium* sp. strain TF04) using lactic acid and acetic acid at concentrations of 15% and 20%, respectively. Using the *Bifidobacterium* sp. strain TF04 antimicrobial agent as a control, the inhibitory activity was measured, with results presented in Figure 5.

The results showed that neither the acetic acid nor the lactic acid treatment zones produced any inhibition zones, whereas the control group exhibited clear inhibition zones. This indicates that lactic acid and acetic acid, adjusted to the same pH as the antimicrobial agent, did not demonstrate significant inhibitory effects against *E. coli*, *S. aureus*, or *S. epidermidis*. These findings preliminarily exclude organic acids as the primary antimicrobial substances in the *Bifidobacterium* sp. strain TF04 antibacterial agent.

#### 3.4.2. Exclusion of Hydrogen Peroxide

To further characterize the antimicrobial substances produced by *Bifidobacterium* sp. strain TF04, the potential contribution of hydrogen peroxide (H_2_O_2_) was examined. Since certain probiotic strains secrete H_2_O_2_ as an antimicrobial compound, catalase—an enzyme that catalyzes the decomposition of H_2_O_2_—was added to the cell-free culture supernatant (CFS) of *Bifidobacterium* sp. strain TF04. If the observed antibacterial activity were exclusively mediated by H_2_O_2_, catalase treatment would abolish its effects, leading to a loss of inhibitory activity.

The experimental results (Table 1) demonstrated that the inhibition zone diameters of the catalase-treated *Bifidobacterium* sp. strain TF04 antimicrobial agent against *E. coli*, *S. aureus*, and *S. epidermidis* were 12.74 ± 0.28 mm, 14.75 ± 0.21 mm, and 17.67 ± 0.19 mm, respectively. No statistically significant reduction was observed compared to the untreated control group. These findings preliminarily exclude hydrogen peroxide as the primary antimicrobial substance responsible for the observed inhibitory activity.

#### 3.4.3. Protease Sensitivity of Bacteriostatic Agent

An additional established mechanism by which probiotics exert antimicrobial effects is through the production of bacteriocins [24]. Bacteriocins are ribosomally synthesized antimicrobial peptides capable of inhibiting both Gram-positive and Gram-negative bacterial growth, thereby eliminating susceptible strains [25]. Notably, bifidobacteria have been demonstrated to synthesize bacteriocins. For example, *Bifidobacterium* BB04 isolated from the intestinal tract of long-lived elderly individuals can produce the broad-spectrum bacteriocin Bifidocin A [26], whereas Bifidin I, a bacteriocin purified from *Bifidobacterium infantis* BCRD14602 fermentation broth, also exhibits broad-spectrum antimicrobial activity [27].

To identify the antimicrobial substances produced by *Bifidobacterium* sp. strain TF04, the antimicrobial agents secreted by this strain were subjected to treatment with various proteases, including pepsin, trypsin, alkaline protease, proteinase K, and papain. Untreated *Bifidobacterium* sp. strain TF04 antimicrobial agents served as the control group. Antimicrobial activity was evaluated by measuring the inhibition zone diameters using the agar well diffusion method.

**Figure 6:**
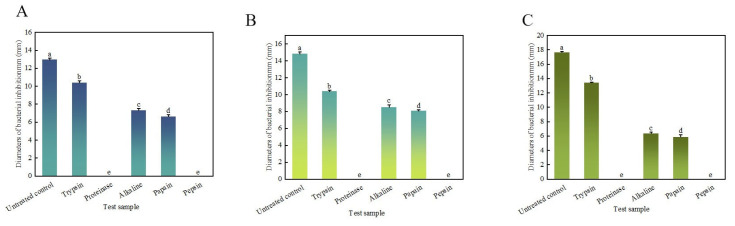
Effect of protease on activity of antibacterial agents. (**A**) shows the inhibitory activity of the bacteriostatic agent on *E. coli* under different protease treatments. (**B**) shows the inhibitory activity of the bacteriostatic agent against *S. aureus* under different protease treatments. (**C**) shows the inhibitory activity of antibacterial agents against *S. epidermidis* under different protease treatments.

The antibacterial results of different treatment groups are shown in Table 1 and Figure 6.

After treatment with different proteases, the antibacterial properties of the bacteriostatic agents exhibited varying degrees of decline. Notably, the antibacterial activity was completely lost after treatment with Pepsin and Protease K. Specifically, the antibacterial activity of Papain treatment on *E. coli*, *S. aureus*, and *S. epidermidis* decreased to 51.3%, 54.4%, and 33.3%, respectively. After treatment with Alkaline protease, the antibacterial activity against *E. coli*, *S. aureus*, and *S. epidermidis* decreased to 56.6%, 57.3%, and 36.0%, respectively. Notably, bacteriostatics exhibited the lowest sensitivity to Trypsin. Following Trypsin treatment, the antibacterial activity against three indicator bacteria still retained above 70%.

### 3.5. Stability Test of Bacteriostatic Agent

#### 3.5.1. Acid-Base Stability of Bacteriostatic Agent

**Figure 7:**
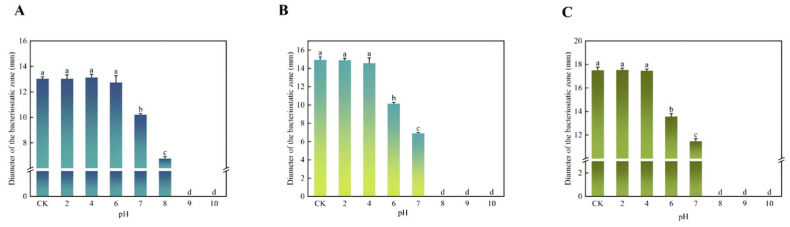
Effect of pH on the activity of antibacterial agents. (**A**) shows the inhibitory activity of the bacteriostatic agent on *E. coli* under different pH. (**B**) shows the inhibitory activity of the bacteriostatic agent against *S. aureus* under different pH. (**C**) shows the inhibitory activity of antibacterial agents against *S. epidermidis* under different pH.

The antibacterial properties of the bacteriostatic agents at different pH were tested. The experimental results are depicted in Figure 7.

The bacteriostatic agent produced by *Bifidobacterium* sp. strain TF04 has high bacteriostatic activity in the pH range of 2–4, while the bacteriostatic performance decreases rapidly or even disappears completely with a further increase in pH. *Bifidobacterium* sp. strain TF04 has the widest range of antibacterial activity against *E. coli*, maintaining substantial antibacterial properties in a wide range of acidic and weak alkaline environments. While it retains inhibitory activity against *S. aureus* and *S. epidermidis* under acidic conditions, this activity is lost when pH > 7.

#### 3.5.2. Thermal Stability and Ultraviolet Stability of Bacteriostatic Agent

**Figure 8:**
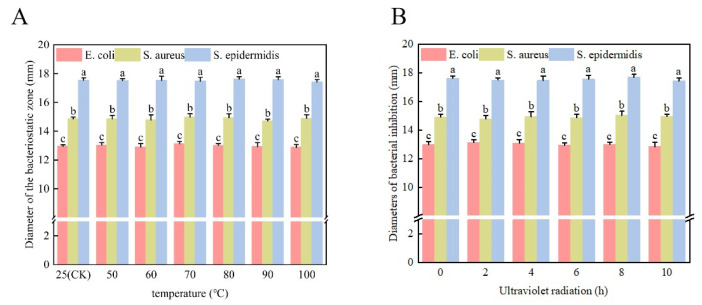
Thermal stability and UV sensitivity testing of bacteriostatic agents. (**A**) shows thermal stability of bacteriostatic agents. (**B**) shows UV sensitivity of bacteriostatic agents.

The antibacterial agent was subjected to thermal stability testing at 50 °C, 60 °C, 70 °C, 80 °C, 90 °C, and 100 °C for 30 min each. Additionally, ultraviolet stability was assessed by exposing the bacteriostatic agent to ultraviolet light for 0 h, 2 h, 4 h, 6 h, 8 h, and 10 h, respectively. The experimental outcomes are illustrated in Figure 8.

After different temperature treatment, the bacteriostatic performance of the bacteriostatic agent exhibited no significant alterations, highlighting the high thermal stability of the bacteriostatic agent derived from *Bifidobacterium* sp. strain TF04. Similarly, the bacteriostatic effect remained consistent after exposure to ultraviolet light irradiation, indicating the stability of the bacteriostatic agent under ultraviolet light.

### 3.6. Probiotic Analysis

#### 3.6.1. Resistance to Low pH and Bile Salts

The growth and metabolism of microorganisms are affected by pH. Given that the pH range of human gastric juice typically spans from 1.5 to 3.0, strains must possess adequate acid tolerance to effectively colonize the gut and manifest their advantageous effects. Consequently, pH tolerance testing was conducted on *Bifidobacterium* sp. strain TF04 to assess its viability under varying pH conditions.

**Figure 9:**
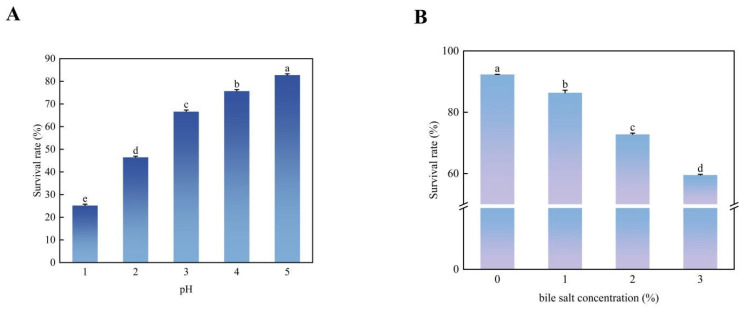
Tolerance analysis of *Bifidobacterium* sp. strain TF04. (**A**) shows the survival rate of *Bifidobacterium* sp. strain TF04 bacteria under different pH treatments. (**B**) shows the survival rate of *Bifidobacterium* sp. strain TF04 bacteria under different bile salt concentration treatments.

The experimental results (Figure 9) revealed a notable increase in the survival rate of *Bifidobacterium* sp. strain TF04 (*p* > 0.05) with escalating pH levels from pH 1 to 5. The survival rate of the strains was 25.65% in the acidic environment of pH = 1. Interestingly, the survival rate of the strains reached more than 65% when the pH was three and notably reached 82.75% when pH was five. Under acidic environment, the strains still had a high survival rate, indicating the acid tolerance of *Bifidobacterium* sp. strain TF04.

Bile salt tolerance stands as a critical attribute for probiotics as they necessitate a certain level of tolerance to bile salts to successfully colonize the intestinal tract. With the increase in bile salt concentration, the survival rate of *Bifidobacterium* sp. strain TF04 decreased gradually. Notably, the survival rate of *Bifidobacterium* sp. strain TF04 was still more than 55% under the condition of 3% bile salt, indicating robust growth capabilities in environment with high bile salt content (Figure 9).

#### 3.6.2. Auto-Aggregation Ability and Hydrophobicity

The self-aggregation capacity of probiotic strains correlates with their cellular adhesion potential. An enhanced self-aggregation phenotype facilitates effective colonization of host cell surfaces, particularly intestinal epithelia. This robust adhesion confers resistance to fluid shear forces, thereby potentiating probiotic efficacy [28].

**Figure 10:**
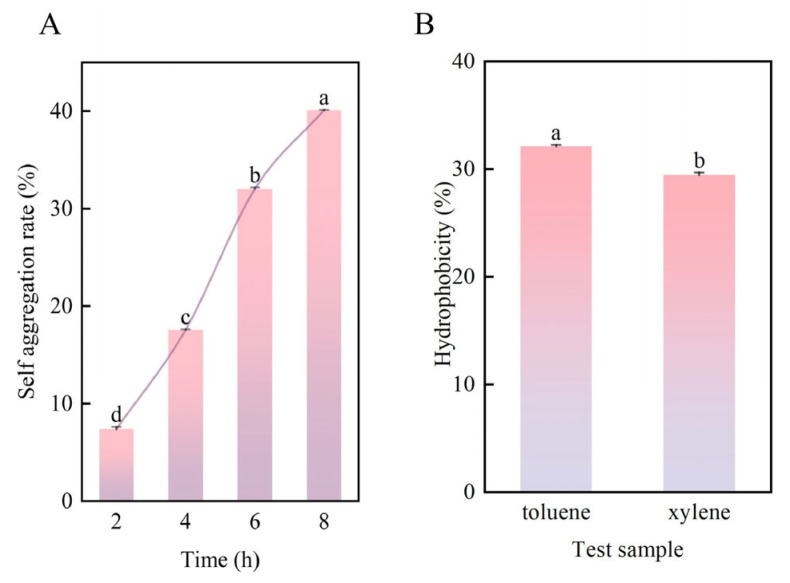
Auto-aggregation ability and hydrophobicity of *Bifidobacterium* sp. strain TF04. (**A**) shows the auto-aggregation ability of *Bifidobacterium* sp. strain TF04. (**B**) shows the hydrophobicity of *Bifidobacterium* sp. strain TF04 bacteria under different bile salt concentration treatments.

The auto-aggregation rate of *Bifidobacterium* sp. strain TF04 is shown in Figure 10. Initially, the strain exhibited low self-aggregation ability at 2 h (7.37%). Subsequently, from 2 h to 8 h, the self-aggregation rate of *Bifidobacterium* sp. strain TF04 steadily increased. Notably, *Bifidobacterium* sp. strain TF04 demonstrated substantial self-aggregation exceeding 40.10% after 8 h.

Studies have confirmed that the higher the surface hydrophobicity of the strain, the stronger its interaction with epithelial cells, consequently enhancing its adhesion capabilities [29,30]. Therefore, the hydrophobicity of *Bifidobacterium* sp. strain TF04 was tested and analyzed in this study, with the experimental outcomes presented in Figure 10. The experimental results show that the hydrophobicity of *Bifidobacterium* sp. strain TF04 to toluene and xylene exceeds 30%.

#### 3.6.3. Safety Assessment of *Bifidobacterium* sp. Strain TF04

**Figure 11:**
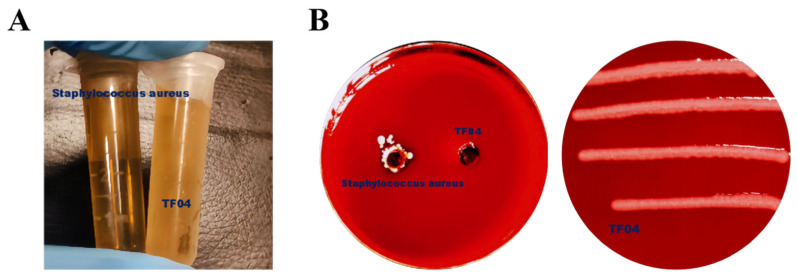
Safety assessment of *Bifidobacterium* sp. strain TF04. (**A**) Gelatinase activity. (**B**) Hemolytic activity and incubation of *Bifidobacterium* sp. strain TF04 in Columbia blood plate.

The safety of *Bifidobacterium* sp. strain TF04 was evaluated with *Staphylococcus aureus* as a positive control, as depicted in Figure 11. *Bifidobacterium* sp. strain TF04 has high safety, without hemolysis and gelatinase activity.

#### 3.6.4. Antioxidant Activity of *Bifidobacterium* sp. Strain TF04

The DPPH (2,2-diphenyl-1-picrylhydrazyl) free radical scavenging assay represents a widely utilized and validated method for assessing antioxidant activity. This stable nitrogen-centered organic free radical serves as an established indicator for antioxidant evaluation. To quantify the antioxidant potential of the studied strain, we assessed the DPPH scavenging capacity of both the bacterial suspension and the fermentation supernatant.

**Figure 12:**
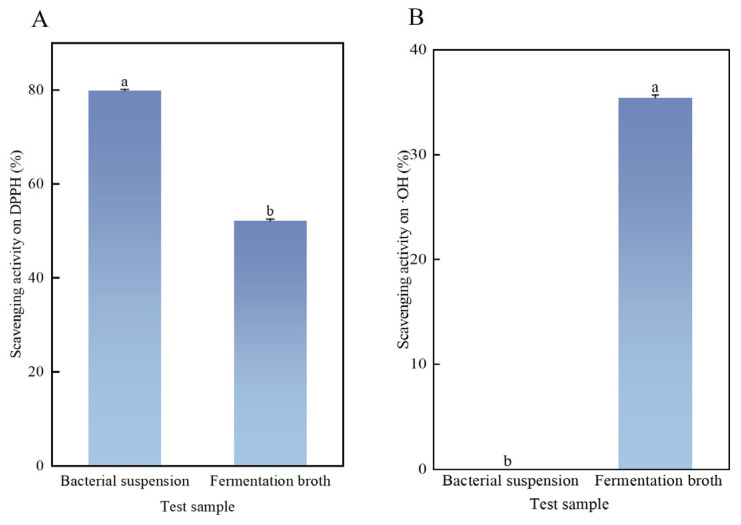
Antioxidant activity of *Bifidobacterium* sp. strain TF04. (**A**) Scavenging activity on DPPH (**B**) Scavenging activity on •OH.

The experimental results indicate that the DPPH free radical scavenging capability of the *Bifidobacterium* sp. strain TF04 bacterial suspension surpasses that of fermentation broth. The difference between the two is significant. The scavenging rate of bacterial suspension on DPPH free radical was about 80%, while the fermentation broth exhibited a scavenging rate of around 50% (Figure 12).

The hydroxyl radical (•OH), regarded as the most reactive oxygen species (ROS), exhibits exceptional cytotoxicity among free radicals. This highly reactive molecule readily interacts with essential biomolecules, including nucleic acids, proteins, and carbohydrates, inducing oxidative damage, cellular structural, functional disruption, and subsequent metabolic dysregulation that may initiate various pathological conditions. In this current investigation, the •OH scavenging capacity of *Bifidobacterium* sp. strain TF04 was evaluated using both bacterial suspension and fermentation broth, with the experimental results illustrated in Figure 12.

The *Bifidobacterium* sp. strain TF04 fermentation broth exhibited a notable scavenging rate of hydroxyl radicals, while the bacterial suspension had no hydroxyl radical scavenging ability. Specifically, the scavenging rate of hydroxyl radicals by *Bifidobacterium* sp. strain TF04 fermentation broth was 35.16%.

## 4. Discussion

Various factors, encompassing physical, chemical, and biological aspects, pose risks to food safety. And the harm of microorganisms to food safety is particularly significant. The growth and reproduction of pathogens in food play a key role in the spread of foodborne diseases. Additionally, microbial-induced food spoilage results in huge economic losses. Therefore, the significance of safe bacteriostatic agents in the domain of food preservation is paramount. Probiotics and their by-products offer a viable approach to impede the growth of deleterious microorganisms, thereby extending the shelf life of food products [31]. In this study, probiotics with antibacterial activity were screened out, with the aim of assessing their applicability in food preservation. This approach seeks to enhance food safety measures, extend the shelf life of food products, and promote consumer safety and well-being.

In this study, the fermentation supernatant of probiotics preserved in the laboratory was evaluated, followed by the identification of strains exhibiting significant antibacterial properties for the formulation of bacteriostatic agents. Subsequently, an exploration of potential active components in the bacteriostatic agents was conducted, alongside assessments of the stability of the produced bacteriostatic agents under diverse conditions and a systematic evaluation of the probiotic characteristics of the selected strains.

Studies have confirmed that probiotics, such as *Bifidobacterium*, can produce antibacterial compounds to cause harm to pathogenic bacteria [32,33]. Physiologically active substances produced by probiotics with bactericidal properties include hydrogen peroxide, short-chain fatty acids, extracellular polysaccharides, and antimicrobial peptides, among others. In this study, to elucidate the potential components of the active antibacterial substances, comparative experiments were conducted using acetic acid and lactic acid at identical pH levels as the bacteriostatic agent. This approach aimed to exclude the possibility of organic acids as antibacterial active substances. In addition, the bacteriostatic agent after catalase treatment still had strong bacteriostatic effect, indicating that the bactericidal activity was not primarily attributed to the hydrogen peroxide generated through strain metabolism. However, following treatment with various proteases, the antibacterial properties of the agents have decreased to varying degrees, with some agents exhibiting a complete loss of inhibitory function. It is speculated that the antibacterial active substances of the bacteriostatic agent may be polypeptides or proteins produced during the metabolic process. Nevertheless, the precise molecular structures and mechanisms of these potential antibacterial proteins or peptides remain uncharacterized, and their identification as functional protein components has not yet been achieved. Consequently, comprehensive characterization of these antimicrobial compounds using proteomics coupled with bioactivity-guided isolation should be prioritized in subsequent investigations.

To broaden the applicability range and guarantee stable bacteriostatic performance of the bacteriostatic agent, apart from exhibiting robust bacteriostatic efficacy, the stability of the agent is a critical parameter in practical utilization. This study explored the stability of the bacteriostatic properties of the agent under various protease treatments. While proteases have the capability to hydrolyze the active components within the bacteriostatic agents, there remains a degree of resilience against specific proteases. Studies have confirmed that some small molecule polypeptides exhibit resistance to specific proteases. For example, bacteriocin LS2 isolated from *Lactobacillus* salivarius BGHO1 has good antibacterial properties and tolerance to proteases after passage and expression in Chlamydomonas reinhardtii cells [34]. Plantaricin LD1, obtained from *Lactobacillus plantarum* LD1, retained partial antibacterial activity even after protease treatment [35].

Numerous previous studies have observed that bacteriocins generally demonstrate higher activity in acidic environments compared to alkaline conditions. Prior research investigating the pH stability of bacteriocins with antimicrobial activity produced by Pediococcus demonstrated that these compounds maintain stable inhibitory activity across a broad pH range [36]. Similarly, Nisin produced by *Lactococcus lactis* exhibits maximum stability at pH 3–3.5, with gradual stability reduction as pH increases [37]. Lasik-Kurdyś et al. [38] conducted systematic characterization of antimicrobial peptides derived from *Oenococcus oeni* isolated from wine, revealing that these peptides maintained stable antibacterial effects within the pH range of 2.0–8.0, but experienced rapid attenuation of inhibitory activity when environmental pH exceeded 8.0—a finding that correlates strongly with the pH sensitivity characteristics observed in the current study. The pH stability profile of Nisin from *Streptococcus lactis* also exhibited striking resemblance to that of the bacteriostatic agent examined in this research [39]. Structure–activity relationship study at the molecular level suggests that under alkaline conditions, the β-sheet structural domain of antimicrobial peptides undergoes irreversible folding, leading to exposure of hydrophobic cores and alterations in the spatial configuration of functional epitopes [40]. This mechanism elucidates the molecular basis of pH-dependent antimicrobial activity regulation. Based on this, we hypothesize that the inactivation of *Bifidobacterium* sp. strain TF04-derived bacteriostatic agents under alkaline conditions may result from alkaline hydrolysis of antimicrobial peptide molecules [36]. An alternative hypothesis suggests that electrostatic interactions under alkaline conditions may induce protein denaturation, resulting in subsequent activity loss. This mechanism could potentially explain the observed disappearance of antimicrobial activity in *Bifidobacterium* sp. strain TF04 under alkaline conditions [41].

Generally, the thermal stability of proteins is inversely correlated with their molecular weight [42]. Therefore, it can be speculated that the antimicrobial substances produced by *Bifidobacterium* sp. strain TF04 during metabolism may be small-molecule proteins or amino acid-derived peptides with high heat resistance. Additionally, the stability of proteins under high temperatures may be influenced by multiple factors, such as hydrogen bonds, salt bridges, proline residues, and polar surface residues [43]. These structural features could also contribute to the excellent thermal stability of the bacteriostatic agent from *Bifidobacterium* sp. strain TF04. In recent years, a multitude of antimicrobial peptides with bacteriostatic activity have been identified, the majority of which display robust thermal stability. For instance, a study characterizing an antimicrobial peptide isolated from *Brevibacterium linens* 7WMA2 demonstrated that it preserved its antimicrobial activity even after exposure to 121 °C, highlighting its remarkable heat resistance [44]. Similarly, bacteriocin LS2, produced by *Lactobacillus salivarius* BGHO1 derived from human oral microbiota, is a thermostable peptide with antimicrobial properties. It maintained its antibacterial activity even after autoclaving at 121 °C for 15 min or boiling for 1 h [45], corroborating the findings of this study. Such high-level stability enables the bacteriostatic agent to be employed in a broader spectrum of practical scenarios and ensures its sustained efficacy during use.

Although ultraviolet radiation can induce structural modifications in proteins, potentially leading to functional impairment or degradation, the antimicrobial activity of the *Bifidobacterium* sp. strain TF04-derived agent remained unaffected in this study. Such findings are not unprecedented. Wang [46] reported similar UV stability in the bacteriostatic agent produced by *Lactobacillus rhamnosus* L57, where UV irradiation had no discernible impact on its inhibitory efficacy, corroborating the results of the present investigation. Furthermore, a study on the physicochemical properties of bacteriocins from *Lactobacillus paracasei* revealed that short-term UV exposure did not significantly alter antimicrobial activity, and even prolonged irradiation and marginally reduced efficacy while maintaining inhibitory activity above 93% [47]. These collective findings underscore the robustness of certain bacteriostatic agents, including that of *Bifidobacterium* sp. strain TF04, under UV stress.

**Figure 13:**
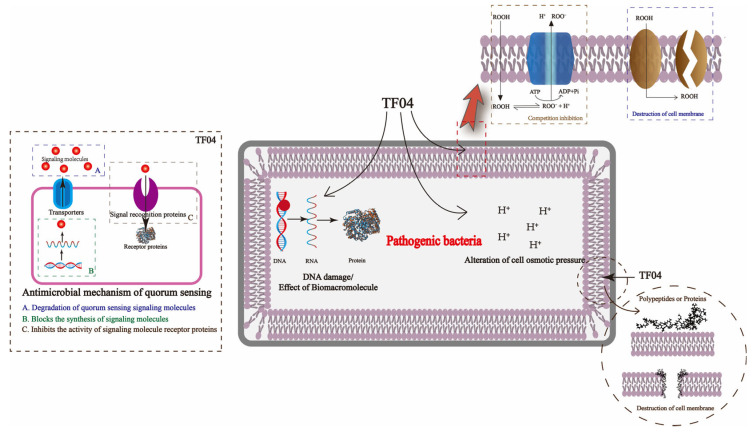
Antibacterial mechanism of *Bifidobacterium* sp. strain TF04.

The mechanism by which *Bifidobacterium* sp. strain TF04 inhibits bacteria was hypothesized by considering the pertinent characteristics of the bacteriostatic agent. The fermentation broth of *Bifidobacterium* sp. strain TF04 exhibits an acidic nature, suggesting the production of organic acids during its fermentation process, including acetic acid, lactic acid, fatty acids with a high carbon count, and phenyl lactic acid. These organic acids have the potential to suppress the proliferation of detrimental microorganisms through competitive inhibition, enhancing the permeability of the bacterial cell membrane, altering the intracellular osmotic balance, and hindering the synthesis of macromolecules. The bacteriostatic properties of the bacteriostat synthesized by *Bifidobacterium* sp. strain TF04 were significantly diminished following exposure to protease, leading to the hypothesis that its primary bacteriostatic agents could be peptides or proteins. Bacteriocins are antimicrobial peptides or proteins synthesized by bacteria as a byproduct of their metabolic processes, exhibiting potent antibacterial and antifungal properties [48]. Furthermore, the majority of bacteriocins possess membrane-active capabilities, facilitating the creation of pores within the cell membrane through binding and integration. This action results in the efflux of intracellular materials and culminates in the demise of the cell [49]. Furthermore, *Bifidobacterium* sp. strain TF04 can also potently suppress the proliferation of pathogenic microorganisms and the establishment of biofilms by leveraging microbial community sensing. This process involves microorganisms generating a category of signaling molecules, termed autoinducers, whose concentration correlates with the density of the microbial population. Upon reaching a specific threshold, these signaling molecules trigger the expression of pertinent genes by activating and binding to their respective receptor proteins [50]. The spoilage potential, genetic potential, virulence, and biofilm formation capabilities of certain spoilage and pathogenic bacteria are linked to quorum sensing [51]. It has been discovered that the spoilage capabilities of microorganisms responsible for the deterioration of dairy, meat, aquatic, and vegetable products are governed by this group sensing mechanism [52]. Consequently, *Bifidobacterium* sp. strain TF04 may suppress the proliferation and pathogenicity of spoilage or pathogenic bacteria by interfering with or inhibiting bacterial quorum sensing. The proposed mechanism underlying the antibacterial activity of *Bifidobacterium* sp. strain TF04 is summarized below (Figure 13).

As a natural bacteriostatic agent or food preservative, its safety in practical applications must also be carefully evaluated. The bacteriostatic agents employed in this study are produced through probiotic fermentation, providing significant health benefits by effectively inhibiting the proliferation of pathogenic bacteria. Thus, the probiotic characteristics of *Bifidobacterium* sp. strain TF04 were investigated. For bacterial strains to be considered probiotics, they must possess several essential characteristics, including the ability to remain viable and metabolically active under gastrointestinal stress conditions [53]. In vitro assessments, such as tests evaluating acid and bile salt tolerance, can serve as valuable indicators of a strain’s viability in the host environment [54]. The *Bifidobacterium* sp. strain TF04 can survive in low pH and high bile salt concentration in vitro experiments, suggesting its potential to colonize the intestinal tract and fulfill a probiotic role. Song et al. conducted a study screening lactic acid bacteria from diverse sources, assessing their tolerance to low pH and bile salts. With the exception of *Lactobacillus acidophilus* M23, all strains exhibited low pH tolerance in the presence of pepsin, with half of the strains displaying high tolerance to bile salt [55]. A previous study has indicated that bifidobacteria have stronger tolerance to pH in the presence of pepsin. Pepsin can protect bifidobacteria in pH environment by reducing the super disproportionation of bifidobacteria cells, a process related to H+-ATP activity [56].

The successful colonization of intestinal epithelial cells serves as a primary criterion for evaluating probiotic strains [57]. The physicochemical properties of microbial surfaces play a crucial regulatory role in their host colonization capacity, among which bacterial adhesion represents a key determinant [58]. The auto-aggregation capacity and hydrophobicity of strains are intrinsically associated with their adhesive properties, as strains exhibiting higher auto-aggregation capacity and hydrophobicity demonstrate stronger interaction forces with epithelial cells, thereby enhancing their ability to withstand fluid shear forces and achieve superior colonization [59]. As a critical physicochemical parameter, the interfacial hydrophobic characteristics of bacterial cells significantly influence the nonspecific adhesion efficacy between probiotics and the intestinal mucosal interface [60]. Mechanistic analysis has revealed that the hydrophobic properties displayed by bacterial strains exhibit a positive correlation with their biointerface affinity. This characteristic significantly enhances microbial adhesion efficiency by reinforcing intermolecular interactions between bacterial cells and host cells. Strains exhibiting high hydrophobicity demonstrate superior mucosal binding affinity, indicating their potential for enhanced intestinal colonization [30].

Cell surface hydrophobicity (CSH) represents a critical screening indicator for potential adhesive probiotics [61]. The research team led by Shekh et al. assessed the surface hydrophobicity of 10 *Lactobacillus* strains, revealing significant inter-strain variability (*p* < 0.05) in interfacial affinity, with hydrophobicity values ranging from 10 to 37%. This observed strain-specific disparity suggests that CSH may function as a key physicochemical parameter for probiotic screening [62]. In a parallel study, Zhao et al. isolated 24 *Bifidobacterium longum* strains from healthy infant feces and measured their CSH, reporting a hydrophobicity range of 21.70–69.67% in xylene solutions [63]. To enhance the probiotic efficacy of bifidobacteria by optimizing CSH, Yolmeh et al. [64] employed a response surface methodology to investigate the effects of ohmic heating conditions on *B. animalis* subsp. *lactis.* Under optimal processing parameters, CSH was significantly improved, reaching 44.82%. The findings of this study align with previously reported interfacial behaviors of probiotics. *Bifidobacterium* sp. strain TF04 exhibited notably high surface hydrophobicity in toluene/xylene systems, a characteristic that likely enhances its mucoadhesive potential. This pronounced interfacial hydrophobicity endows *Bifidobacterium* sp. strain TF04 with distinct advantages in maintaining gut microbiota homeostasis and exerting probiotic functions, thereby providing a theoretical foundation for developing probiotic-based antimicrobial agents.

In screening for potential gastrointestinal probiotics, a study has revealed that 85% of tested *Lactobacillus* strains exhibit auto-aggregation capability [65]. Fonseca et al. [66] systematically evaluated the probiotic properties of lactic acid bacteria based on stress resistance, safety, and functionality. Among the tested strains, *Lactobacillus paracasei* CCMA0504 and *L. paracasei* CCMA0505 demonstrated the highest auto-aggregation values (45.46% and 52.66%, respectively). Similarly, Wang et al. [67] isolated *Lactobacillus plantarum* AR113 and *Lactobacillus plantarum* AR501 from traditional Chinese pickles and evaluated their auto-aggregation capacity. After 5 h of incubation, the auto-aggregation levels reached 30.10% and 29.29%, respectively. Consistent with these findings, *Bifidobacterium* sp. strain TF04 demonstrated strong auto-aggregation ability, a trait that could promote intestinal colonization and thereby enhance its probiotic efficacy. This observation corroborates prior studies, underscoring the significance of auto-aggregation as a critical factor in probiotic strain selection.

Systematic assessment of cytolytic toxin secretion capacity represents a critical component in the functional validation of probiotic candidates. Current pathomechanistic studies demonstrate that Gram-positive bacterial α-hemolysins induce host cell membrane potential through formation of transmembrane β-barrel pores, resulting in osmolytic lysis [3,68]. In contrast, β-hemolysins exert their cytotoxic effects via sphingomyelinase-mediated hydrolysis of structural phospholipids, initiating cascading membrane destabilization. Due to the well-established correlation between cytolytic activity and pathogenicity, the International Scientific Association for Probiotics and Prebiotics (ISAPP, 2020) consensus guidelines explicitly require that all candidate strains be subjected to standardized hemolysis assays as part of the essential biosafety verification protocols [3]. Gelatinase, as a member of the metalloproteinase superfamily, has been demonstrated by multi-omics studies to possess pathological significance: this enzyme compromises basement membrane integrity through specific cleavage of type IV collagen α1 chains, facilitating tumor cell extravasation and metastatic microenvironment formation [69]. Moreover, gelatinolytic activity shows significant correlation with matrix metalloproteinase-9 expression levels [70]. Consequently, the FAO/WHO (2002) [71] Probiotic Production Guidelines strictly require standardized gelatin liquefaction assays for rigorous strain screening to eliminate potential oncogenic risks. Thus, *Bifidobacterium* sp. strain TF04, which demonstrates neither gelatinase nor hemolytic activities, fulfills the safety prerequisites for potential probiotic applications.

Probiotics have demonstrated efficacy in mitigating and preventing various health disorders, with their beneficial effects potentially mediated by antioxidant properties [72]. Substantial evidence indicates that *Bifidobacterium* strains can modulate host physiological and metabolic homeostasis through their potent antioxidant activity. For instance, *Bifidobacterium longum* subsp. *longum* CCFM752 has been shown to attenuate hypertension and aortic lesions in rat models [73], while its culture supernatant significantly suppresses the elevation of O_2_^−^ and H_2_O_2_ levels in A7R5 smooth muscle cells derived from rat thoracic aorta [46]. Furthermore, increased intestinal colonization by *Bifidobacterium* significantly reduces cardiac risk factors and atherogenic indices in broiler chickens [74]. Consequently, antioxidant capacity serves as a critical biomarker for evaluating the probiotic potential of microbial strains.

The DPPH radical scavenging assay has been widely adopted as a standardized method for evaluating the antioxidant capacity of bioactive compounds. As a prototypical nitrogen-centered stable free radical, DPPH• possesses exceptional thermodynamic stability due to its unique triphenylmethyl conjugated structure, which facilitates single electron transfer (SET)-mediated redox reactions with hydrogen-donating compounds [75]. Its ethanolic solution exhibits a characteristic violet color in the visible spectrum, with a distinct absorption peak at 517 nm. Upon introduction of electron-donating substances, the electronic transition of the radical is markedly altered, resulting in solution decolorization [76]. Given its reliability, the DPPH scavenging model has been endorsed by international antioxidant assessment standards as a quantitative spectrophotometric method (UV-Vis) for determining the electron-donating capacity of biological samples. Accordingly, this study evaluated the DPPH radical scavenging activity of both cell suspensions and fermented supernatants of *Bifidobacterium* sp. strain TF04. Substantial experimental evidence demonstrates that both fermented supernatants and cell suspensions of bifidobacteria exhibit significant DPPH radical scavenging activity. In a representative study, Wang et al. [44] analyzed the free radical scavenging efficacy of human-derived *Bifidobacterium* longum subsp. longum isolates. Their findings revealed that metabolite fractions (fermented supernatants) displayed remarkable DPPH scavenging activity, with all tested strains except 1b63-15 and 2b3-6 exceeding 50% scavenging efficiency. Particularly noteworthy was strain 1b38-1, which demonstrated exceptional scavenging capacity (91.81%). In contrast, the whole-cell systems (cell suspensions) exhibited significant strain-dependent heterogeneity in antioxidant performance, as evidenced by a 56.7 percentage point range in scavenging efficiency (20.9–77.6%). This inter-strain variability may be attributed to differences in cell membrane permeability and heterogeneous expression of intracellular antioxidant enzymes.

Hydroxyl radicals, recognized as the most cytotoxic reactive oxygen species (ROS), induce irreversible oxidative damage through multiple reaction pathways including hydrogen abstraction, electron transfer, and electrophilic addition. These highly reactive radicals can oxidize DNA bases, modify protein thiol groups, and initiate lipid peroxidation chain reactions, ultimately leading to mitochondrial membrane potential collapse and increased nuclear DNA double-strand breaks [77]. Such oxidative damage is strongly associated with the pathogenesis of neurodegenerative disorders and atherosclerosis. To assess the antioxidative potential of *Bifidobacterium* sp. strain TF04, we evaluated the hydroxyl radical scavenging capacity of both its cell suspension and fermented supernatant. These experimental results with previous reports documenting strain-specific variations in hydroxyl radical scavenging capacity between cellular and extracellular components. Zhao et al. [63] reported that *Bifidobacterium longum* subsp. *longum* K5 displayed 58.76% and 25.57% scavenging activity for cell suspension and fermented supernatant, respectively. Similarly, *Bifidobacterium longum* BB-12 showed scavenging rates of 39.00% (cell suspension) and 21.02% (fermented supernatant). These comparative data highlight the significant interspecies and intraspecies variability in antioxidative mechanisms among bifidobacteria.

Extensive data are available regarding the antioxidant mechanisms of *Bifidobacterium*. Iron-binding sites have been identified across various *Bifidobacterium* species, indicating their capacity to chelate ferrous ions, thereby inhibiting their catalytic role in oxidative reactions [78]. Furthermore, a study has demonstrated that cells incubated with the culture supernatant of *B. longum* exhibit upregulated translation levels of proteins associated with catalase synthesis, enhancing intracellular catalase activity. Catalase, which degrades hydrogen peroxide, has been widely recognized as a pivotal enzyme in the antioxidant defense system [46]. *Bifidobacterium* strains can also mitigate oxidative stress by activating critical pathways within the antioxidant defense network [79]. Additionally, *Bifidobacterium* synthesizes reduced glutathione to preserve epithelial redox homeostasis while concomitantly secreting water-soluble B vitamins (e.g., folate) that function as essential methyl donors. These vitamins modulate protein expression via epigenetic regulation, thereby enhancing the antioxidant efficacy of high-density lipoprotein. The acidic metabolites produced by *Bifidobacterium* metabolism contribute to maintaining a low intestinal pH, limiting the proliferation of pathogenic bacteria and consequently mitigates oxidative stress responses [80]. In summary, the antioxidant activity of *Bifidobacterium* is achieved through metal ion sequestration, activation of antioxidant enzyme systems, and production of antioxidant metabolites that modulate the gut microbiota. This study preliminarily confirms the antioxidant effects of *Bifidobacterium* sp. strain TF04 through in vitro antioxidant activity assays.

## 5. Conclusions

The aim of this study was to screen probiotics with high bacteriostatic activity from 23 strains of probiotics kept in the laboratory. *Bifidobacterium* sp. strain TF04 was obtained through screening processes. Its fermentation broth had strong inhibitory effect on *Escherichia coli*, *Staphylococcus aureus*, and *Staphylococcus epidermidis*. At the same time, an in-depth analysis of the bacteriostatic properties was conducted. The bacteriostatic agent showed a strong stability, maintaining its bacteriostatic activity in a wide pH range. Additionally, it demonstrated a certain degree of resistance to protease hydrolysis and exhibited favorable thermal and UV stability. Moreover, *Bifidobacterium* sp. strain TF04 showed a high level of survival in simulated gastrointestinal environments, including low pH and bile salt environments. *Bifidobacterium* sp. strain TF04 also showed a high level of auto-aggregation, surface hydrophobicity and antioxidant activity. In vitro experiments confirmed the absence of hemolytic and gelatinase activities, underscoring its safety profile. In summary, the findings of this study underscore the secure and efficacious production of bacteriostatic agents by *Bifidobacterium* sp. strain TF04, concurrently showcasing its noteworthy probiotic characteristics.

## Figures and Tables

**Table 1:** Enzyme susceptibility of inhibitory active substances of *Bifidobacterium* sp. strain TF04.

Test Sample	Zone Diameter (mm)		
	*Escherichia coli*	*Staphylococcus aureus*	*Staphylococcus epidermidis*
Untreated control	12.94 ± 0.18 ^a^	14.81 ± 0.22 ^a^	17.62 ± 0.17 ^a^
Catalase	12.74 ± 0.28 ^a^	14.75 ± 0.21 ^a^	17.67 ± 0.19 ^a^
Trypsin	10.38 ± 0.23 ^b^	10.37 ± 0.15 ^b^	13.41 ± 0.07 ^b^
Proteinase K	—	—	—
Alkaline protease	7.32 ± 0.21 ^c^	8.49 ± 0.31 ^c^	6.34 ± 0.19 ^c^
Papain	6.64 ± 0.17 ^d^	8.06 ± 0.13 ^d^	5.87 ± 0.30 ^d^
Pepsin	—	—	—

Note: Different superscript letters (a, b, c, d) within the same column indicate statistically significant differences between treatments (*p* < 0.05, one-way ANOVA followed by a Tukey post-hoc test).

## Data Availability

Sequence data that support the findings of this study have been deposited in the National Center for Biotechnology Information with the GenBank accession number PQ637525. The strain was preserved in the China General Microbiological Culture Collection Center, CGMCC. The conservation number is CGMCC No. 32629.
